# Supplementary material for: Microbial Regulation of Enteric Eosinophils and Its Impact on Tissue Remodeling and Th2 Immunity
Source: Front Immunol. 2020 Feb 13;11:155. doi: 10.3389/fimmu.2020.00155 (PMC7033414; doi:10.3389/fimmu.2020.00155)
Supplement: Supplementary file 1 [file Data_Sheet_1.docx]

**Supplementary Figure 1**. SPF and GF mice were intragastrically sensitized to peanut (PN) and serum levels of PN-specific IgE (A) and IgG1 (B ) were determined by ELISA. Data from 6-8 mice (A, B) from 2 experiments represented as mean ± SEM, *P<0.05.
